# Impact of SGLT2 inhibitors on lower limb complications: a mendelian randomization perspective

**DOI:** 10.3389/fphar.2024.1401103

**Published:** 2024-09-17

**Authors:** Baixing Chen, Mingling Huang, Bin Pu, Hang Dong

**Affiliations:** ^1^ Department of Development and Regeneration, KU Leuven, Leuven, Belgium; ^2^ Department of Diabetes, Central Clinical School, Monash University, Melbourne, VIC, Australia; ^3^ Department of Orthopedics, Suining City Traditional Chinese Medicine Hospital, Affiliated with North Sichuan Medical College, Sichuan, China; ^4^ The First Affiliated Hospital, Guangzhou University of Chinese Medicine, Guangzhou, China

**Keywords:** SGLT2 inhibition, osteomyelitis, peripheral artery disease, ulcers, cellulitis, mendelian randomization

## Abstract

**Background:**

While Sodium-glucose cotransporter 2 (SGLT2) inhibitors are effective in managing diabetes and reducing cardiovascular risk, concerns about their association with lower limb complications, including, osteomyelitis, ulcers, and peripheral artery disease (PAD), persist. This study employs Mendelian Randomization (MR) to assess the causal relationship between SGLT2 inhibitors and these lower limb safety outcomes.

**Methods:**

A two-sample drug-target MR approach was used, complemented by a one-sample MR and genetic association analysis. Six SNPs were selected as instrumental variables to proxy the effect of SGLT2 inhibition. Primary outcomes were major limb safety outcomes, including osteomyelitis, lower limb ulcers, PAD, and cellulitis. The primary analytical method was the generalized inverse variance-weighted (IVW) approach, along with several sensitivity analyses.

**Results:**

The MR analysis indicated no significant causal association between genetically proxied SGLT2 inhibition and most of the studied lower limb safety outcomes. However, a significant association with PAD was observed, necessitating careful interpretation due to discrepancies between IVW and MR-Egger results. Sensitivity analyses supported these findings, showing little evidence of heterogeneity or directional pleiotropy.

**Conclusion:**

This study suggests that SGLT2 inhibitors may not be significantly associated with an increased risk of most lower limb safety outcomes, including osteomyelitis, lower limb ulcers, and cellulitis, in patients with type 2 diabetes. However, the complex relationship with PAD highlights the need for further research. These findings contribute to the understanding of the safety profile of SGLT2 inhibitors, supporting their continued use in diabetes management while underlining the importance of continuous safety monitoring.

## Introduction

Sodium–glucose cotransporter 2 (SGLT2) inhibitors, including medications like canagliflozin, dapagliflozin, and empagliflozin, have gained widespread approval as antihyperglycemic drugs. They are known for their effectiveness in reducing blood sugar levels and decreasing the risk of cardiovascular issues in diabetic patients (Scheen, 2020; Xu B. et al., 2022). While these medications offer significant advantages, in 2017, the FDA announcement highlighted an increased risk of lower limb amputations, particularly with canagliflozin usage (UFaD; Chang et al., 2018; Neal et al., 2017; Lee, 2017). Despite the removal of this warning in 2020 after further evaluation of its benefits, the continued mention of amputation risks associated with SGLT2 inhibitors in the warnings and precautions section of their prescribing information emphasizes persistent concerns.

Furthermore, lesser-explored complications, involving lower limb ulcers, osteomyelitis, and other related infections pose a rapidly increasing threat, especially for patients with type 2 diabetes, potentially heightening the risk of amputation (Chang et al., 2018; Neal et al., 2017; Nani et al., 2023; Custodio et al., 2020; Dicembrini et al., 2019). Current research shows mixed results, complicating our understanding of these negative outcomes. One retrospective cohort study highlighted an increased risk of osteomyelitis, peripheral artery disease (PAD), and ulcers (Chang et al., 2018). On the other hand, a meta-analysis of randomized controlled trials (RCTs) involving type 2 diabetes patients treated with SGLT2 inhibitors found a generally neutral impact on osteomyelitis and PAD, but a link to local ulcers was noted (Nani et al., 2023). The repercussions of these adverse effects go beyond the immediate health issues, influencing patient quality of life, clinical decision-making, and public health strategies.

Considering the complexities and potential biases in observational studies, our research employs Mendelian Randomization (MR) as a reliable method to explore the cause-and-effect relationship between SGLT2 inhibitors and lower limb safety outcomes (Dai et al., 2023). MR uses specific genetic variants linked to SGLT2 inhibitors as instrumental variables, enabling a simulation of random assignment of individuals to varying exposure levels, akin to a RCT (Sekula et al., 2016). This methodology increases the accuracy of our results by reducing confounding factors and pleiotropy, thus providing a more nuanced understanding of the safety profile of SGLT2 inhibitors, particularly regarding lower limb complications in type 2 diabetes patients (Burgess et al., 2023).

With the rising incidence of diabetes and the escalating use of SGLT2 inhibitors in its management, thoroughly investigating potential risks to lower limbs is essential for responsible and informed medical care (Khouri et al., 2018). Our study, based on MR, seeks to clarify the intricate link between SGLT2 inhibitors and lower limb safety outcomes, focusing on the genetic factors that contribute to these worrying complications.

## Materials and methods

### Study design

**FIGURE 1:**
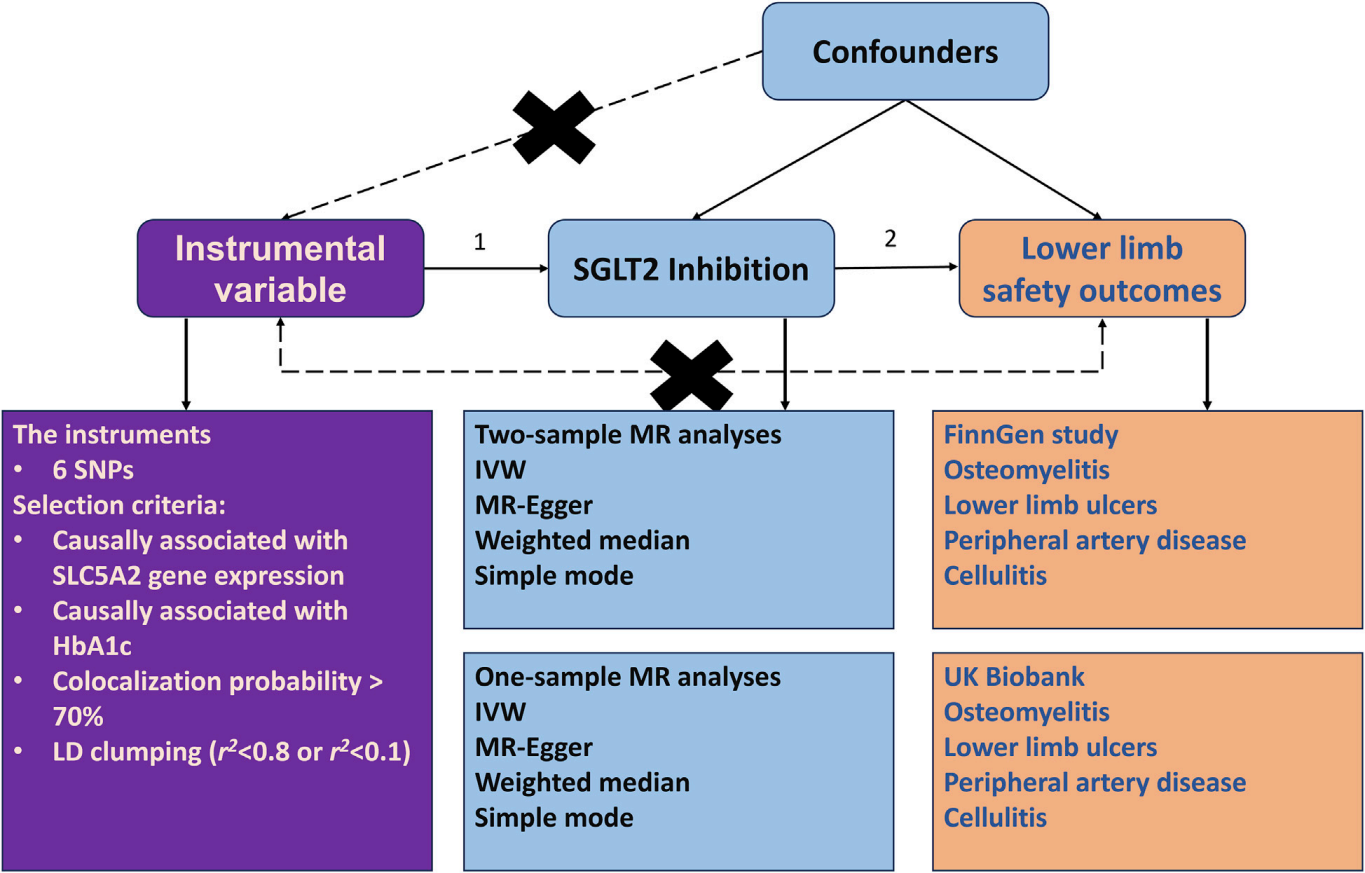
Study design. The research involved two-sample Mendelian randomization (MR) analyses to explore the impact of SGLT2 inhibition on lower limb safety outcomes. The figure at the top was employed to illustrate the theoretical associations among genetic variants (SNPs), SGLT2 inhibitors (exposure), and lower limb safety outcomes. This graphical depiction accounts for the potential influence of unobserved confounding variables. In this diagram, solid arrows denote permissible relationships between these variables, reflecting known or hypothesized causal connections. Conversely, dashed lines are utilized to indicate relationships that are proscribed. These prohibited connections are crucial for selected SNPs to meet the criteria for their validity as an instrumental variable. The figure at the bottom shows six single-nucleotide polymorphisms (SNPs), serving as instrumental variables, which were carefully chosen as proxies for the effect of SGLT2 inhibition. Lower limb safety outcomes, encompassing osteomyelitis, lower limb ulcers, peripheral artery disease and cellulitis were identified as the targeted outcomes. Summary data for both exposure and outcomes were sourced from pertinent meta-analyses of genomewide association analyses. The primary method for estimating the causal effect on selected outcomes was the generalized inverse variance-weighted approach. The study incorporated several sensitivity analyses. Additionally, one-sample MR and genetic association analyses were conducted, utilizing individual-level data from the UK Biobank to validate the findings from the two-sample MR. Abbreviations: IVW = inverse variance weighted; LD = linkage disequilibrium; SGLT2 = sodium-glucose cotransporter 2; SNP = single-nucleotide polymorphism.

Figure 1 illustrates the comprehensive design of our study. Our goal was to evaluate the potential causal effects of SGLT2 inhibition on osteomyelitis and other critical limb safety issues using a dual-method approach: a two-sample drug-target MR combined with a one-sample MR. First, the SNPs, selected based on their association strength and relevance, act as proxies for SGLT2 inhibition for the next step. We then focused on outcomes such as osteomyelitis, as well as other significant limb safety outcomes like lower limb ulcers, PAD, and cellulitis. The primary method of analysis was the inverse variance-weighted (IVW) method, which was reinforced by various sensitivity analyses, including MR-Egger, weighted median, simple mode, and weighted mode approaches, to verify the strength of our results. Following this, we performed a one-sample MR analysis within the UK Biobank for these outcomes. This step was intended to support and authenticate the initial findings from the two-sample MR approach.

### Genetic associations with SGLT2 inhibition

To develop genetic instruments that act as surrogates for the long-term glucose-lowering effects of SGLT2 inhibition, we implemented a four-stage instrument selection process, as previously described (Dai et al., 2023; Xu M. et al., 2022). Initially, we identified genetic variants associated with the mRNA expression of the SLC5A2 gene, leveraging data from the Genotype-Tissue Expression (GTEx) project (Consortium, 2020) and the eQTLGen Consortium (Vosa et al., 2021). This step focused on identifying potential functional genes of SGLT2 inhibitors (refer to Supplementary Table S1). Next, the connection between each SLC5A2 variant and HbA1c levels was assessed, which was indicative of the glucose-reducing impact of SGLT2 inhibition. Variants with a region-wide link to HbA1c were selected based on data from a subset of UK Biobank participants of European descent without diabetes (association *p*-value = 1 × 10^−4^) (see Supplementary Table S1) (Elsworth et al., 2020). The third phase involved validating whether SLC5A2 and HbA1c shared a common causal variant through genetic colocalization analysis. This method, a bivariate Bayesian model, estimated the likelihood that SLC5A2 expression and HbA1c levels in circulation were influenced by the same causal variant within the SLC5A2 region (Walker et al., 2020). Lastly, a standard clumping process was executed (applying a correlation threshold <0.8 to exclude highly correlated variants). The efficacy of the genetic variants as predictors was then evaluated using F statistics, focusing on their statistical power for each exposure tested. Following these rigorous steps, six genetic variants strongly linked to SGLT2 inhibition through HbA1c were selected as genetic instruments for the MR analysis (refer to Supplementary Table S2).

### Study outcomes

The main focus of our study was on significant limb safety outcomes. For the subsequent MR analysis, we employed summary statistics from relevant genome-wide association studies (GWAS) pertaining to these outcomes. We sourced the GWAS data for limb safety outcomes like osteomyelitis, lower limb ulcers, PAD, and cellulitis from the FinnGen study, which was used in the two-sample drug-target MR analyses (Kurki et al., 2023). Additionally, GWAS data from the UK Biobank was employed for a one-sample MR analysis (Backman et al., 2021) (refer to Supplementary Table S2).

### Statistical analysis

#### MR analyses of SGLT2 inhibition and limb safety outcomes

We gathered and analyzed summary data of six instrumental SNPs’ genetic associations from GWAS relevant to our study. To align the effects of an SNP on both the exposure and the outcome, harmonization procedures were put into place before carrying out causal estimations. We employed the IVW method (Burgess et al., 2017) to bolster the analysis power. This method accounted for the correlation among the six genetic predictors of SGLT2 inhibition, allowing for a more lenient clumping threshold. An linkage disequilibrium (LD) matrix for each pair of variants was obtained from the 1000 Genomes dataset, and the IVW method was applied to assess the MR effect while incorporating the LD matrix of the genetic variants.

### Validation of MR assumptions and sensitivity analyses

The study findings were presented following the STROBE-MR (Strengthening the Reporting of Mendelian Randomization Studies) guidelines (Skrivankova et al., 2021). Three essential MR assumptions were validated through various sensitivity analyses. The relevance assumption was verified by evaluating the strength of the genetic predictors using R^2^ and F-statistics, with an F-statistic above 10 indicating a robust defense against weak instrument bias. The exclusion restriction assumption underwent scrutiny through diverse sensitivity analyses, including MR-Egger regression, weighted median analysis, and both simple and weighted mode analyses. Cochran’s Q test was utilized to determine instrument heterogeneity. For the one-sample MR analyses using UK Biobank data, where both the exposures and outcomes are measured in the same individuals, we employed the two-stage least squares (2SLS) regression method.

Statistical analyses were performed using the ‘TwoSampleMR’ packages. We applied Bonferroni corrections for multiple tests, setting adjusted significance thresholds at 0.013 (0.05/4). In terms of ethical considerations, the FinnGen and UK Biobank study received informed consent from participants and was approved by its institutional review board.

## Result

### Genetic predictors of SGLT2 inhibition strength

Supplementary Table S1 detailed the features of the genetic instruments (rs4488457, rs8057326, rs11865835, rs9930811, rs34497199, and rs35445454) utilized as proxies for SGLT2 inhibition (see Supplementary Table S2). These instruments demonstrated substantial robustness (F-statistics = 24.1), suggesting a minimal chance of weak instrument bias.

### Effect of genetically proxied SGLT2 inhibition on limb safety outcome

**FIGURE 2:**
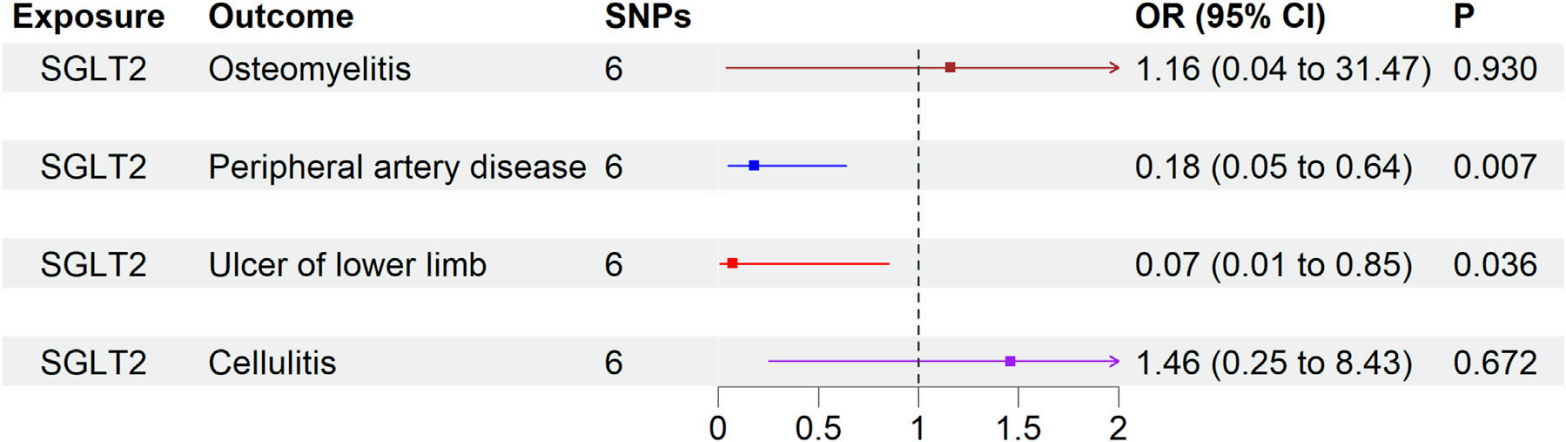
The causal effect of genetically proxied SGLT2 inhibition on lower limb safety outcome. Data are presented as the lower limb safety outcome via SGLT2 inhibition estimated by the generalized inverse variance weighted method. *p* < 0.013 is considered significant difference. CI = confidence interval; IV = instrumental variable; SGLT2 = sodium-glucose cotransporter 2; SNP = single-nucleotide polymorphism.

Genetically proxied SGLT2 inhibition, utilizing six SNPs as instruments for IVW analysis, did not show significant associations with osteomyelitis (OR 1.16, 95% CI 0.04–31.47, *p* = 0.930), PAD (OR 0.18, 95% CI 0.05–0.64, *p* = 0.007), lower limb ulcers (OR 0.07, 95% CI 0.006–0.85, *p* = 0.036), or cellulitis (OR 1.46, 95% CI 0.25–8.43, *p* = 0.672) (Figure 2).

### Sensitivity analyses

Additional sensitivity analyses, including MR-Egger, weighted median, simple mode and weighted mode analyses (Supplementary Table S3), consistently provided little evidence supporting an association between genetically proxied SGLT2 inhibition and the mentioned outcomes. Notably, for PAD, MR-Egger analysis showed a different direction of association compared to the IVW approach. The observed discrepancies between the IVW and MR-Egger methods may imply uncertainty regarding the role of SGLT2 inhibition as a protective or risk factor. While the IVW approach suggested a potential protective effect against the outcome, the conflicting direction indicated by the MR-Egger method indicated a cautious interpretation of SGLT2’s influence, necessitating further investigation to clarify its impact on limb safety outcomes. Heterogeneity testing using the Cochran Q test for IVW and pleiotropy testing using the MR-Egger intercept term suggested minimal evidence of heterogeneity or directional pleiotropy (Supplementary Table S3).

### One-sample MR analysis in UK biobank

In the one-sample MR analysis conducted in the UK Biobank (Supplementary Table S4), the SGLT2 inhibition constructed from six SNPs did not show a significant association with any limb safety outcome.

## Discussion

Our study used MR to investigate the causal relationship between SGLT2 inhibitors and lower limb safety outcomes, specifically addressing the concerns raised by previous observational studies and the FDA’s safety communication regarding the use of SGLT2 inhibitors. The key findings suggest that while there is no significant association with most lower limb complications such as osteomyelitis, ulcers, and cellulitis, the connection with PAD observed requires cautious interpretation. The discrepancy between the IVW and MR-Egger results suggests that this association may be influenced by pleiotropic effects, which could operate through mechanisms other than direct SGLT2 inhibition.

The global recognition of gliflozins’ benefits in patients with type 2 diabetes mellitus and their cardio- and nephroprotective advantages in broader patient populations is well-established (Margonato et al., 2021; Granata et al., 2022). These drugs have been shown to decrease glycated hemoglobin, enhance major metabolic parameters, and significantly reduce all-cause and cardiovascular mortality (Teo et al., 2021). However, alongside these benefits, it is crucial to continuously evaluate their safety profile, especially considering concerns about adverse lower limb events.

Our MR findings contribute to the ongoing discussion about the safety of SGLT2 inhibitors, particularly in the context of lower limb complications such as amputations. Previous literature, including RCTs and observational studies, has produced mixed results regarding the association between SGLT2 inhibitors and lower limb adverse events (Lindbom et al., 1988). A cohort study involving commercially insured patients, reflective of real-world scenarios, demonstrated that users of SGLT-2 inhibitors exhibited higher rates of PAD, osteomyelitis, and lower limb ulcers when compared to individuals using metformin, sulfonylureas, or thiazolidinediones. However, notably, such associations were not observed when compared to new users of dipeptidyl peptidase-4 (DPP-4) inhibitors (Chang et al., 2018). In contrast, a comprehensive meta-analysis of 42 RCTs with 29,491 patients on SGLT2 inhibitors and 23,052 in control groups revealed no significant association with osteomyelitis and PAD, but a heightened risk of ulcers (Nani et al., 2023). This highlights the need for personalized medical approaches that consider individual risk factors and comorbid conditions when prescribing these medications.

In understanding the pathophysiological mechanisms behind the risks associated with SGLT2 inhibitors, it’s crucial to consider the broader context of type 2 diabetes mellitus. Patients with this condition inherently face an elevated risk of ulcers and infections, which can lead to more severe complications such as amputations (Armstrong et al., 2017; Jeffcoate and Harding, 2003). For outcomes like PAD and ulcer of lower limb, our MR study suggests a more complex relationship than what has been reported in some RCTs and observational studies (Brownrigg et al., 2013). Genetic variants mimicking SGLT2 inhibition represent lifelong effects, yet they may not reflect the short-term clinical impacts or the gradual development of conditions like PAD and lower limb ulcers. The inconsistent result between MR-Egger and IVW for PAD may arise due to the limited number of included SNPs, potentially leading to less accurate estimates in the MR, which performs best with a larger number of genetic variants (Bowden et al., 2015). Another explanation for this is possible unaddressed pleiotropy or biases in our instrumental variables. Despite no significant heterogeneity or pleiotropy affecting our instrumental variables as indicated by the tests, these findings call for a cautious approach to clinical application. Future research endeavors should consider refining genetic instruments, exploring broader datasets, and extending follow-up durations to provide a more nuanced understanding of the relationship between SGLT2 inhibition and PAD.

Regarding osteomyelitis, the evidence from the FDA Adverse Event Reporting System (FAERS) database introduces an intriguing perspective (Zhao et al., 2023). It suggests that exposure to SGLT2 inhibitors, particularly canagliflozin, may be a primary cause of osteomyelitis in diabetic patients. However, the lack of association with other widely used SGLT2 inhibitors, such as dapagliflozin and empagliflozin, emphasizes the need for a granular examination of individual drugs within the SGLT2 inhibitor class (Mascolo et al., 2022). This study, when compared with observational studies, illuminates the methodological strengths of MR analysis. Genetic variants that mimic SGLT2 inhibition reflect the lifelong effects of these inhibitors on the expression levels of SLC5A2. However, the magnitude of these effects may not accurately represent the short-term impacts of SGLT2 inhibitors (Klen and Dolzan, 2021). Therefore, MR analysis is more valuable for determining the direction of potential causal effects rather than for quantifying their magnitude. Meanwhile, we used the largest data on osteomyelitis currently available and employed various sensitivity analyses to assess the robustness of our findings. While observational studies may be susceptible to potential confounders and draw relative risks, the MR study, leveraging genetic variants as instrumental variables, is less likely to be affected by confounders. This allows for a more direct evaluation of the causal effect of SGLT2 inhibition on adverse outcomes in the lower extremities, enhancing the reliability of our findings.

Several limitations should be acknowledged in interpreting our study findings. Firstly, our MR analysis estimated the effect of SGLT2 inhibition based on on-target reductions in HbA1c levels rather than the direct effects of SGLT2 inhibitors. This assumption hinges on the idea that the effect of SGLT2 inhibition on HbA1c levels proportionally represents its overall impact, which may not entirely align with the actual mechanism of SGLT2 inhibition. Additionally, the definitions used for outcomes, particularly ulcer of lower limb, may exhibit variations across different datasets and studies. The absence of standardized definitions introduces variability, potentially affecting result comparability. Moreover, our research did not extend to evaluating the risk of lower limb complications associated with SGLT2 inhibitors in non-diabetic populations, such as individuals with heart failure or chronic kidney disease who do not exhibit diabetic symptoms. This exclusion is notable given emerging evidence suggesting differential effects of SGLT2 inhibitors in non-diabetic cohorts, including potential cardiovascular and renal benefits that might influence the overall risk profile of lower limb complications. Therefore, caution is warranted in generalizing our findings to diverse patient groups. Further replication studies in diverse ethnic groups are needed to validate these findings and ensure broader applicability. Considering these limitations is crucial for a nuanced interpretation of our study results. Future research endeavors could benefit from addressing these limitations and incorporating complementary approaches to enhance the depth and reliability of our understanding regarding the potential risks associated with SGLT2 inhibitor use in individuals with diabetes.

In conclusion, our study suggests that the use of SGLT2 inhibitors may not be significantly associated with an increased risk of most lower limb safety outcomes. These findings contribute to the ongoing discourse on the safety and efficacy of SGLT2 inhibitors and support their continued use in diabetes management. Future research should continue to explore this area, potentially expanding the scope to include other populations and long-term outcomes, to fully understand the implications of SGLT2 inhibitor use in diabetes care.

## Data Availability

The datasets presented in this study can be found in online repositories. The names of the repository/repositories and accession number(s) can be found in the article/Supplementary Material.
