# Supplementary material for: Impact of SGLT2 inhibitors on lower limb complications: a mendelian randomization perspective
Source: Front Pharmacol. 2024 Sep 17;15:1401103. doi: 10.3389/fphar.2024.1401103 (PMC11442421; doi:10.3389/fphar.2024.1401103)
Supplement: Supplementary file 1 [file Table1.DOCX]

**Supplementary Material**

**Title:** Impact of SGLT2 Inhibitors on Lower Limb Complications: A Mendelian Randomization Perspective

**Authors:** Baixing Chen^1^, Mingling Huang^2^, Bin Pu^3^, Shi Lin^3^, Shaoshuo Li^4^, Hang Dong^5*^

**Supplementary Tables**

**Supplementary Table 1.** Characteristics of genetic variants associated with HbA1c and used as proxies for SGLT2 inhibition in the general population

**Supplementary Table 2.** Data sources

**Supplementary Table 3**. Two-sample MR analyses of genetically proxied SGLT2 inhibition on limb safety outcomes from FinnGee study

**Supplementary Table 4.** One-sample MR analyses of genetically proxied SGLT2 inhibition on limb safety outcomes from UKBiobank study

**Supplementary Table 1.** Characteristics of genetic variants associated with HbA_1c_ and used as proxies for SGLT2 inhibition in the general population

| **Genetic predictors** | **Gene** | **Effect allele/**  **Non-effect allele** | **Effect allele frequency** | **Beta** | **Se** | **P values** | **F statistics** |
| --- | --- | --- | --- | --- | --- | --- | --- |
| rs4488457 | *SLC5A2* | G/T | 0.712 | -0.013 | 0.003 | 2.90E-07 |  |
| rs8057326 | *SLC5A2* | C/T | 0.523 | -0.008 | 0.002 | 2.80E-04 |  |
| rs11865835 | *SLC5A2* | C/T | 0.284 | -0.011 | 0.003 | 1.34E-05 |  |
| rs9930811 | *SLC5A2* | G/A | 0.365 | -0.016 | 0.002 | 8.69E-12 |  |
| rs34497199 | *SLC5A2* | T/C | 0.475 | -0.012 | 0.002 | 5.98E-07 |  |
| rs35445454 | *SLC5A2* | T/C | 0.344 | -0.013 | 0.002 | 1.24E-07 | 24.1 |

Two sets of instruments that proxying SGLT2 inhibition used same instrument selection processes but not same criterion. CI, confidence interval.

**Supplementary Table 2**. Data sources

| **Traits** | **Data source** | **Year** | **Sample size** | **Case** | **Control** | **Population** | **Data sources** |
| --- | --- | --- | --- | --- | --- | --- | --- |
| SGLT2 inhibition | UK Biobank | 2018 | 344,182 | NA | NA | European | https://gwas.mrcieu.ac.uk/datasets/ukb-d-30750_irnt/ |
| Osteomyelitis | FinnGen | 2021 | 210,417 | 842 | 209,575 | European | https://gwas.mrcieu.ac.uk/datasets/ieu-b-4975/ |
| Osteomyelitis | UK Biobank | 2021 | 486.484 | 4,836 | 481,648 | European | https://gwas.mrcieu.ac.uk/datasets/finn-b-M13_OSTEOMYELITIS/ |
| Ulcer of lower limb | FinnGen | 2021 | 209,066 | 1,584 | 207,482 | European | https://gwas.mrcieu.ac.uk/datasets/finn-b-L12_ULCERLOWLIMB/ |
| Ulcer of lower limb | UK Biobank | 2021 | 387,464 | 1,708 | 385,756 | European | https://www.ebi.ac.uk/gwas/studies/GCST90080381 |
| Peripheral artery disease | FinnGen | 2021 | 213,639 | 7,098 | 206,541 | European | https://gwas.mrcieu.ac.uk/datasets/finn-b-I9_PAD/ |
| Peripheral artery disease | UK Biobank | 2021 | 483,078 | 7,114 | 475,964 | European | https://gwas.mrcieu.ac.uk/datasets/ebi-a-GCST90018890/ |
| Cellulitis | FinnGen | 2021 | 211,534 | 3,085 | 208,449 | European | https://gwas.mrcieu.ac.uk/datasets/finn-b-L12_CELLULITIS/ |
| Cellulitis | UK Biobank | 2021 | 486,484 | 12,196 | 474,288 | European | https://gwas.mrcieu.ac.uk/datasets/ieu-b-4970/ |

**Supplementary Table 3.** Two-sample MR analyses of genetically proxied SGLT2 inhibition on limb safety outcomes from FinnGee study

| **Outcomes** | **N participants** | **Methods** | **N snp** | **β（95% CI)** | **P values** | **P-het** | **P-ple** |
| --- | --- | --- | --- | --- | --- | --- | --- |
| Osteomyelitis | 210,417 | IVW | 6 | 0.15 (-3.15, 3.45) | 0.93 | 0.736 | 0.329 |
|  |  | MR Egger | 6 | -9.07 (-25.68, 7.54) | 0.345 | 0.821 |  |
|  |  | Weighted median | 6 | 0.69 (-3.47, 4.85) | 0.745 |  |  |
|  |  | Weighted mode | 6 | 1.18 (-4.2, 6.55) | 0.686 |  |  |
|  |  | Simple mode | 6 | 1.77 (-3.96, 7.51) | 0.571 |  |  |
| Ulcer of lower limb | 209,066 | IVW | 6 | -2.61 (-5.05, -0.17) | 0.036 | 0.414 | 0.169 |
|  |  | MR Egger | 6 | 7.66 (-4.59, 19.92) | 0.288 | 0.697 |  |
|  |  | Weighted median | 6 | -3.63 (-7.00, -0.26) | 0.035 |  |  |
|  |  | Weighted mode | 6 | -3.96 (-9.95, 2.02) | 0.251 |  |  |
|  |  | Simple mode | 6 | -3.85 (-8.91, 1.22) | 0.197 |  |  |
| Peripheral artery disease | 213,639 | IVW | 6 | -1.69 (-2.92, -0.45) | 0.007 | 0.679 | 0.368 |
|  |  | MR Egger | 6 | 1.46 (-4.75, 7.68) | 0.668 | 0.716 |  |
|  |  | Weighted median | 6 | -1.7 (-3.37, -0.03) | 0.046 |  |  |
|  |  | Weighted mode | 6 | -1.79 (-3.88, 0.31) | 0.155 |  |  |
|  |  | Simple mode | 6 | -2.32 (-4.52, -0.12) | 0.093 |  |  |
| Cellulitis | 211,534 | IVW | 6 | 0.38 (-1.37, 2.13) | 0.672 | 0.418 | 0.556 |
|  |  | MR Egger | 6 | 3.39 (-5.99, 12.77) | 0.518 | 0.341 |  |
|  |  | Weighted median | 6 | 0.66 (-1.66, 2.97) | 0.577 |  |  |
|  |  | Weighted mode | 6 | 0.95 (-2.00, 3.89) | 0.556 |  |  |
|  |  | Simple mode | 6 | 0.64 (-2.81, 4.08) | 0.732 |  |  |

BMD, bone mineral density; SNP, single nucleotide polymorphism; IVW, inverse variance weighted; CI, confidence interval; P-het, p value for heterogeneity test; P-ple, p value for pleiotropy test.

**Supplementary Table 4.** One-sample MR analyses of genetically proxied SGLT2 inhibition on limb safety outcomes from UKBiobank study

| **Outcomes** | **N participants** | **Methods** | **N snp** | **β（95% CI)** | **P values** | **P-het** | **P-ple** |
| --- | --- | --- | --- | --- | --- | --- | --- |
| Osteomyelitis | 486.484 | IVW | 6 | 1.39 (-0.047, 2.826) | 0.058 | 0.947 | 0.798 |
|  |  | MR Egger | 6 | 0.41 (-6.775, 7.595) | 0.916 | 0.894 |  |
|  |  | Weighted median | 6 | 1.51 (-0.304, 3.319) | 0.103 |  |  |
|  |  | Weighted mode | 6 | 1.54 (-0.705, 3.788) | 0.236 |  |  |
|  |  | Simple mode | 6 | 1.59 (-0.845, 4.018) | 0.257 |  |  |
| Ulcer of lower limb | 387,464 | IVW | 6 | -0.002 (-0.007, 0.002) | 0.308 | 0.928 | 0.349 |
|  |  | MR Egger | 6 | 0.01 (-0.014, 0.034) | 0.451 | 0.849 |  |
|  |  | Weighted median | 6 | -0.001 (-0.007, 0.005) | 0.712 |  |  |
|  |  | Weighted mode | 6 | -0.001 (-0.008, 0.006) | 0.722 |  |  |
|  |  | Simple mode | 6 | -0.002 (-0.009, 0.006) | 0.71 |  |  |
| Peripheral artery disease | 483,078 | IVW | 6 | -0.55 (-1.763, 0.658) | 0.371 | 0.189 | 0.332 |
|  |  | MR Egger | 6 | 2.98 (-3.414, 9.368) | 0.413 | 0.221 |  |
|  |  | Weighted median | 6 | -0.22 (-1.519, 1.069) | 0.733 |  |  |
|  |  | Weighted mode | 6 | -0.33 (-1.684, 1.02) | 0.651 |  |  |
|  |  | Simple mode | 6 | -0.62 (-2.261, 1.02) | 0.492 |  |  |
| Cellulitis | 486,484 | IVW | 6 | 0.01 (-0.888, 0.918) | 0.974 | 0.88 | 0.958 |
|  |  | MR Egger | 6 | -0.11 (-4.625, 4.404) | 0.964 | 0.779 |  |
|  |  | Weighted median | 6 | -0.03 (-1.168, 1.109) | 0.959 |  |  |
|  |  | Weighted mode | 6 | -0.09 (-1.636, 1.459) | 0.915 |  |  |
|  |  | Simple mode | 6 | -0.13 (-1.696, 1.432) | 0.875 |  |  |

BMD, bone mineral density; SNP, single nucleotide polymorphism; IVW, inverse variance weighted; CI, confidence interval; P-het, p value for heterogeneity test; P-ple, p value for pleiotropy test.
